# Valvular Heart Disease in a Young Israeli Ethiopian Immigrant Population From the Gondar Region With Implications for Rheumatic Heart Disease

**DOI:** 10.3389/fpubh.2018.00130

**Published:** 2018-05-14

**Authors:** Daniel Lyon Fink, Yoram Chaiter, Samuel Menahem, Rivka Farkash, Yossy Machluf

**Affiliations:** ^1^Shaare Zedek Medical Center, Jerusalem, Israel; ^2^Israel Defense Forces Medical Corps, Tel Hashomer, Ramat Gan, Israel; ^3^Monash University, Melbourne, VIC, Australia; ^4^Shamir Research Institute, University of Haifa, Kazerin, Israel

**Keywords:** valvular heart disease, Ethiopia, Gondar, rheumatic heart disease, adolescents

## Abstract

**Background:**

Rheumatic heart disease (RHD) among Ethiopian school children was recently found to be 1.4%. Immigration of the Jewish population from the Gondar region to Israel created an opportunity for further enquiry.

**Methods:**

A cross-sectional study of the cardiac status of 113,671 adolescent recruits aged 16–19 years from the northern district of Israel who completed the medical profiling process over a 22-year period.

**Results:**

140 recruits had a history of rheumatic fever (0.12%), although none from an Ethiopian origin (*n* = 1,719). The prevalence of valvular heart disease clinically and confirmed echocardiographically in Ethiopian recruits was not different from the total population (0.81 and 0.93%, respectively). However, the prevalence was higher in those migrating to Israel in their 13th year or older (2.09%), compared to those migrating at a younger age or born in Israel (0.49%).

**Conclusion:**

The Ethiopian teenage Israeli population from Gondar had a high rate of auscultation positive and echocardiographically confirmed valvular disease that suggested a high rate of RHD (~1.6%), despite no relevant past history. Our findings also suggested that for the younger Ethiopian immigrants or Israeli born subjects of Ethiopian origin, the improved medical care may well reduce the prevalence of valvular heart disease to that of the rest of the local population.

## Introduction

Rheumatic heart disease (RHD), the most common cardiovascular disease worldwide in those under 25 years, often leads to significant potentially preventable morbidity and mortality (1, 2). Acute rheumatic fever (ARF) may result in clinical valvular lesions, especially after clinical and/or subclinical recurrences (1, 2). Not all patients with ARF develop symptoms and not all symptomatic cases are diagnosed. Therefore, controlling RHD at the population level may necessitate a sensitive detection of subclinical disease—nowadays by portable high resolution echocardiography—prior to developing clinical RHD (1). Historically, screening was much less sensitive based on cardiac auscultation with or without confirmatory echocardiography (1, 3–6). A recent systematic review revealed that the health-related burden of RHD has declined worldwide, but high rates of disease persist in some of the poorest regions in the world, including central sub-Sahara Africa (7). Pre-echo screening estimates of RHD prevalence in African children was 5–10 per 1,000 (8–10). Using echocardiographic diagnostic criteria of the World Heart Federation (11) estimates of RHD were 14–31 per 1,000 in Ethiopia (12, 13), and approximately 7, 18, 20, and 34 per 1,000 in Rwanda (14), Uganda and Tanzania (15), South Africa (13), and Malawi (16), respectively. These findings suggested a much greater disease burden than previously suspected.

The rate of RHD by echocardiographic screening in schools in the Gondar region of Ethiopia was at least 1.4% with definite disease and might be up to 2.4% in the teenage population (12). Moreover, a high carrier rate of Group A *Streptococcus*, a risk factor for developing RHD, was found among a healthy school children population of the Gondar region (17). RHD was found to be a major cause of morbidity and early mortality in an inpatient medical ward in Gondar in the third decade of life (18).

The immigration of the Jewish population from the Gondar region to Israel, as part of a general return of many Jewish populations worldwide, provided an opportunity to further explore the possibility of clinically evident RHD-related valvular heart disease in that population.

The aims of the study were to estimate the prevalence of clinically evident valvular heart disease suggesting RHD in adolescents of Ethiopian background who were born in, or immigrated to, Israel from the Gondar region.

## Materials and Methods

The Israeli National Military Service Act requires all 17-year-old Israelis to present themselves to a recruitment center to undergo a thorough medical profiling process.

### Study Population

The study population of this cross-sectional study consisted of conscripts who were born between 1970 and 1993, examined during the years 1987–2010, completed the medical profiling process at the age of 16–19 years, and had valid height and weight measures. Only the northern recruitment center of Israel was chosen due to its stringent assessment and reliability of its medical data (19–22). Further information regarding the study population, the medical process and validation of the data has been reported earlier (23, 24).

### Cardiac Data Collection

All recruits were interviewed to obtain a detailed medical history, including review of documentations from his/her physician. The recruits underwent a thorough physical examination, independently seen by two well trained and experienced physicians. Those recruits with a suggestion of cardiac pathology, based on documentation and/or auscultation, were referred to a cardiologist. Those with abnormal findings on physical examination and/or by electrocardiography were referred for two-dimensional echocardiography and color Doppler interrogation, and where appropriate, cardiac catheterization, computed tomography, or magnetic resonance imaging—all performed at cardiology centers. Documentation of valvular abnormalities which were used as a surrogate marker for RHD, particularly important for those that failed to provide a relevant history, was tabulated. Recruits with a diagnosis of a valvular abnormality included those with RHD and/or congenital heart disease and rarely other causes of acquired heart disease [see definitions and examples in Ref. (24)].

### Statistical Analysis

Prevalence of valvular anomalies was calculated among Ethiopian immigrants from the Gondar region, Israeli born subjects of Ethiopian origin, and the non-Ethiopian population. Univariate analyses included χ^2^ or Fisher’s exact test to compare categorical variables between groups. The statistical tests were two sided (significance: *p*-value <0.05). All analyses were carried out using the SPSS statistical package (version 22.0; IBM Corporation, Armonk, NY, USA).

### Institutional Review Board (IRB) Approval

The study was approved by an IDF IRB ethics (Helsinki) committee (Approval number: #1199-2012).

## Results

The study population included 113,671 conscripts which were screened over a 22-year period and had completed the medical profiling process between 16 and 19 years of age. Of them, 1,719 conscripts were of Ethiopian origin: 1,029 were born in Ethiopia and immigrated to Israel, and 690 were born in Israel of Ethiopian parents.

The prevalence rate of a history of ARF was 0.12% in the study population (140 adolescents), while the prevalence rate of echocardiographically confirmed valvular disease was 0.93% (1,058 adolescents). A history of ARF was only obtained from conscripts of non-Ethiopian origin (0.13%).

**Figure 1:**
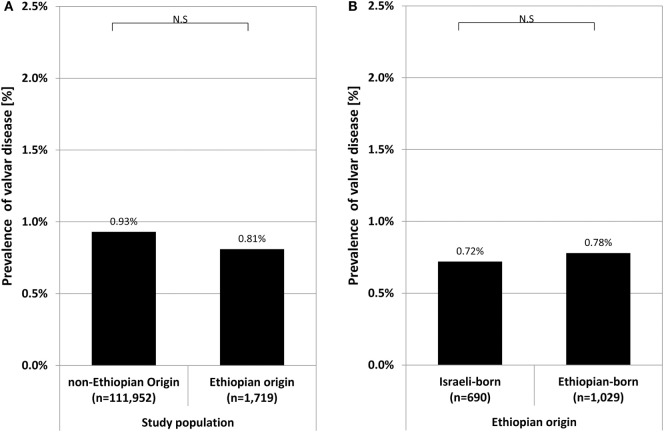
Prevalence rate of valvular heart disease in recruits of Ethiopian origin compared to those of non-Ethiopian origin **(A)**, and in recruits of Ethiopian origin comparing Israeli born to those born in Ethiopia **(B)**. Symbol stands for *p*-value: *p < 0.05; N.S., non significant (*p* ≥ 0.05).

The prevalence of echocardiographically confirmed valvular disease was not significantly different between conscripts of Ethiopian (0.81%, 13 cases) and non-Ethiopian (0.93%, 1,045 cases) origin (Figure 1A). Moreover, the prevalence of valvular disease among conscripts of Ethiopian origin was not different between those born in Ethiopia (0.78%, 8 cases) or in Israel (0.72%, 5 cases) (Figure 1B).

**Figure 2:**
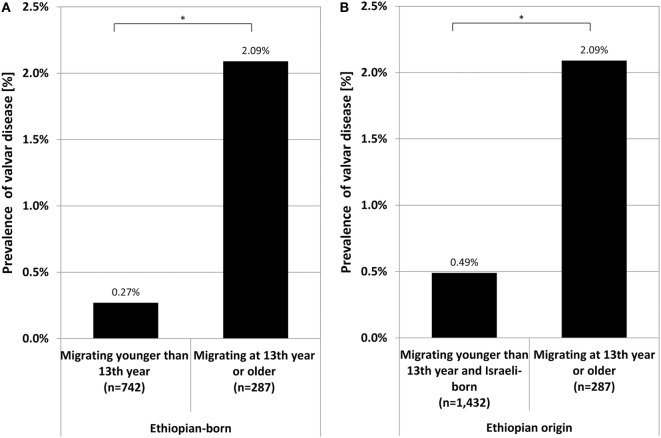
Prevalence of valvular heart disease in Ethiopian-born recruits migrating to Israel in their 13th year or older compared to either those migrating younger solely **(A)** or together with those born in Israel to Ethiopian parents **(B)**. Symbol stands for *p*-value: *p < 0.05; N.S., non significant (*p* ≥ 0.05).

We further investigated the potential relevance of the age of immigration from Ethiopia as to the prevalence of valvular disease. The prevalence among Ethiopians who migrated in their 13th year or older (2.09%, 6 cases) was higher compared to those who migrated at a younger age solely (0.27%, 2 cases, *p* = 0.007, Figure 2A) and remained higher when compared to all other Ethiopians (young immigrants together with those born in Israel) (0.49%, 7 cases, *p* = 0.012, Figure 2B).

If from the prevalence in the older migrating group (2.09%) is subtracted the prevalence in the remaining Ethiopian population (0.49%), a conservative estimated prevalence of auscultation positive additional valvular disease of 1.6% is obtained. This difference in prevalence was likely attributed to RHD in those immigrating as teenagers from the Gondar region.

## Discussion

A history of ARF was not obtained in conscripts of Ethiopian origin, probably due to its under-diagnosis in the African population (25). Therefore, the investigation of valvular abnormalities as a surrogate for RHD was pursued. One would assume that while a small portion of these would relate to a congenital anomaly (see below), most would arise from RHD.

The total Ethiopian Jewish community immigrated to Israel without any selection criteria. The significantly higher prevalence of valvular heart disease among the Ethiopians arriving in Israel when in their 13th year or older compared to the prevalence among the remaining Ethiopians was likely due to RHD as most Ethiopian-born infants/children with serious congenital heart disease would probably have succumbed (26, 27). Most Ethiopian-born conscripts lived in rural villages, suggesting that the majority of sore throats were not treated or had delayed treatment. Furthermore, the late or non-referral to medical centers of infants and children with congenital heart disease led to higher morbidities and increased mortality as reported in the Gondar region of Ethiopia (26) and sub-Saharan Africa (28).

In contrast, Ethiopian children born in Israel or arriving in Israel at a younger age would have better housing and access to the extensive medical services, including: a greater awareness among healthcare professionals ensuring close monitoring and surveillance of relevant cases at the personal and national levels that results in a timely diagnosis and appropriate treatment of streptococcal infections, specialist referral for any with clinical features of ARF followed by confirmation and management of the acute episode with subsequent counseling and the commencement of long-term prophylactic treatment—all anchored in the Israeli health procedures and policies, and afforded nationwide (29–31). That would be especially relevant when no genetic predisposition was found (32, 33), suggesting the overriding importance of environmental factors.

Screening programs in developing countries indicate that the prevalence of RHD increases significantly during the teen years (12). This finding is most likely a consequence of the lag time between the first ARF attack to the development of clinically evident disease, and the greater incidence of having had a first ARF attack in an older cohort. The highest prevalence of RHD in the pediatric population in Africa has been in the teenage years (12, 13) and is consistent with our finding of a significant increase in the rate of cardiac valvular disease, presumed RHD, in those leaving Ethiopia when older.

Our data suggested a very high prevalence of auscultation positive RHD, even though this would be a conservative estimate as we know today that the burden of subclinical disease could be up to 10 times higher. Also, those with significant clinical symptoms from RHD would be less likely to survive and/or would not be included in a generally healthy young population presenting for national service. Our data also suggested further investigation as to the cause of the valvular heart disease in adolescents of Ethiopian origin together with the need for close follow-up.

### Study Limitations

The nature of the study did not permit review of the clinical findings and/or of the echocardiographic data of the affected individuals. In our database, congenital and acquired diseases were grouped together as valvular disease, providing only the categorical diagnosis of isolated valvular disease (without concomitant non-valvular cardiac anomaly). We presumed that the high prevalence of valvular disease among older immigrants from Ethiopia was related to the known high rate of RHD in Ethiopia together with the likelihood that those with serious CHD would not have had the necessary interventions to enable survival. It was also assumed that the Jewish population migrating to Israel was similar constitutionally and was subjected to the same environmental factors as the general Gondar population. We also acknowledge the lack of health data on individuals (including familial history) prior to their immigration from Ethiopia as well as the lack of information on those who did not immigrate, which may have been related to health conditions. These may be related to unobserved or unmeasured confounders of the noted associations.

Of note, 7% of the Ethiopian population in Israel is living in the northern district. Nevertheless, all the Ethiopian population in Israel immigrated from the Gondar region. As they were distributed randomly, one would not expect more than a minor selection bias based on geographic region (northern Israel and other regions).

## Conclusion

We showed that the Israeli teenage-immigrant population from the Gondar region of Ethiopia had auscultation positive and echocardiographically confirmed additional valvular disease of 1.6% which likely represented RHD. This prevalence is high compared to previous reports, despite no apparent past history of ARF. Although the study design did not allow the investigation of genetic susceptibility to RHD, it is reassuring that this large African population, when transferred to a western medical environment early enough, had a prevalence of valvular heart disease similar to the local baseline rate.

## Author Contributions

DF, YM, and YC were involved in all aspects of this study. RF was involved in data analysis and statistics. In addition, all authors conceived the conception and design of this study and were involved in drafting the article, data interpretation, iterative critical revisions for important intellectual content, and final approval of the article. Therefore, each of the authors takes public responsibility for the content, and agrees to be accountable for all aspects of the work including ensuring that questions related to the accuracy or integrity of any part of the work are appropriately investigated and resolved.

## Conflict of Interest Statement

The authors declare that there are no personal, professional, commercial, financial, or any other relationships that could be construed or perceived by the academic or medical communities as representing a potential conflict of interest. The opinions expressed in this manuscript represent the consensus of the authors and do not necessarily reflect the formal (or informal) position of the affiliated institutions.

## Abbreviations

ARF, acute rheumatic fever; RHD, rheumatic heart disease.

## References

[1] Dougherty S Khorsandi M Herbst P. Rheumatic heart disease screening: current concepts and challenges. Ann Pediatr Cardiol (2017) 10(1):39–49. 10.4103/0974-2069.197051 28163427 PMC5241843

[2] Nulu S Bukhman G Kwan GF. Rheumatic heart disease: the unfinished global agenda. Cardiol Clin (2017) 35(1):165–80. 10.1016/j.ccl.2016.08.006 27886787

[3] Ploutz M Lu JC Scheel J Webb C Ensing GJ Aliku T Handheld echocardiographic screening for rheumatic heart disease by non-experts. Heart (2016) 102(1):35–9. 10.1136/heartjnl-2015-308236 26438784

[4] Nascimento BR Nunes MC Lopes EL Rezende VM Landay T Ribeiro AL Rheumatic heart disease echocardiographic screening: approaching practical and affordable solutions. Heart (2016) 102(9):658–64. 10.1136/heartjnl-2015-308635 26891757

[5] Roberts K Colquhoun S Steer A Remenyi B Carapetis J. Screening for rheumatic heart disease: current approaches and controversies. Nat Rev Cardiol (2013) 10(1):49–58. 10.1038/nrcardio.2012.157 23149830

[6] Marijon E Ou P Celermajer DS Ferreira B Mocumbi AO Jani D Prevalence of rheumatic heart disease detected by echocardiographic screening. N Engl J Med (2007) 357(5):470–6. 10.1056/NEJMoa065085 17671255

[7] Watkins DA Johnson CO Colquhoun SM Karthikeyan G Beaton A Bukhman G Global, regional, and national burden of rheumatic heart disease, 1990–2015. N Engl J Med (2017) 377(8):713–22. 10.1056/NEJMoa1603693 28834488

[8] Mirabel M Celermajer DS Ferreira B Tafflet M Perier MC Karam N Screening for rheumatic heart disease: evaluation of a simplified echocardiography-based approach. Eur Heart J Cardiovasc Imaging (2012) 13(12):1024–9. 10.1093/ehjci/jes077 22518053

[9] Oli K Porteous J. Prevalence of rheumatic heart disease among school children in Addis Ababa. East Afr Med J (1999) 76(11):601–5. 10734517

[10] Abdel-Moula AM Sherif AA Sallam SA Mandil AM Kassem AS Zaher SR. Prevalence of rheumatic heart disease among school children in Alexandria, Egypt: a prospective epidemiological study. J Egypt Public Health Assoc (1998) 73(3–4):233–54. 17219923

[11] Remenyi B Wilson N Steer A Ferreira B Kado J Kumar K World Heart Federation criteria for echocardiographic diagnosis of rheumatic heart disease – an evidence-based guideline. Nat Rev Cardiol (2012) 9(5):297–309. 10.1038/nrcardio.2012.7 22371105 PMC5523449

[12] Yadeta D Hailu A Haileamlak A Gedlu E Guteta S Tefera E Prevalence of rheumatic heart disease among school children in Ethiopia: a multisite echocardiography-based screening. Int J Cardiol (2016) 221:260–3. 10.1016/j.ijcard.2016.06.232 27404686

[13] Engel ME Haileamlak A Zuhlke L Lemmer CE Nkepu S van de Wall M Prevalence of rheumatic heart disease in 4720 asymptomatic scholars from South Africa and Ethiopia. Heart (2015) 101(17):1389–94. 10.1136/heartjnl-2015-307444 26076935

[14] Mucumbitsi J Bulwer B Mutesa L Ndahindwa V Semakula M Rusingiza E Prevalence of rheumatic valvular heart disease in Rwandan school children: echocardiographic evaluation using the World Heart Federation criteria. Cardiovasc J Afr (2017) 28:1–8. 10.5830/CVJA-2017-007 28252675 PMC5730679

[15] Moloi AH Mall S Engel ME Stafford R Zhu ZW Zuhlke LJ The health systems barriers and facilitators for RHD prevalence: an epidemiological meta-analysis from Uganda and Tanzania. Glob Heart (2017) 12(1):5–15.e3. 10.1016/j.gheart.2016.12.002 28302546

[16] Sims Sanyahumbi A Sable CA Beaton A Chimalizeni Y Guffey D Hosseinipour M School and Community Screening shows Malawi, Africa, to have a high prevalence of latent rheumatic heart disease. Congenit Heart Dis (2016) 11(6):615–21. 10.1111/chd.12353 27029239

[17] Abdissa A Asrat D Kronvall G Shitu B Achiko D Zeidan M Throat carriage rate and antimicrobial susceptibility pattern of group A Streptococci (GAS) in healthy Ethiopian school children. Ethiop Med J (2011) 49(2):125–30. 21796912

[18] Melka A. Rheumatic heart disease in Gondar College of Medial Sciences Teaching Hospital: socio-demographic and clinical profile. Ethiop Med J (1996) 34(4):207–16. 9164036

[19] Chaiter Y Machluf Y Pirogovsky A Palma E Yona A Shohat T Quality control and quality assurance of medical committee performance in the Israel Defense Forces. Int J Health Care Qual Assur (2010) 23(5):507–15. 10.1108/95268621080000418 20845680

[20] Chaiter Y Pirogovsky A Palma E Yona A Machluf Y Shohat T Medical quality control in conscription centers – ten years of activity. J Israel Mil Med (2008) 5(2):75–9.

[21] Machluf Y Navon N Yona A Pirogovsky A Palma E Tal O From a Quality Assurance and Control System for Medical Processes, Through Epidemiological Trends of Medical Conditions, to a Nationwide Health Project. Modern Approaches To Quality Control. Eldin AB, editor. Rijeka, Croatia: InTech (2011). p. 259–82.

[22] Machluf Y Pirogovsky A Palma E Yona A Navon A Shohat T Coordinated computerized systems aimed at management, control, and quality assurance of medical processes and informatics. Int J Health Care Qual Assur (2012) 25(8):663–81. 10.1108/09526861211270622 23276061

[23] Machluf Y Fink D Farkash R Rotkopf R Pirogovsky A Tal O Adolescent BMI at Northern Israel: from trends, to associated variables and comorbidities, and to medical signatures. Medicine (Baltimore) (2016) 95(12):e3022. 10.1097/MD.0000000000003022 27015176 PMC4998371

[24] Fink DL Machluf Y Farkash R Weisz G Pirogovsky A Tal O Cardiac anomalies and associated comorbidities in a large adolescent population. Int J Adolesc Med Health (2017). 10.1515/ijamh-2017-0020 28614051

[25] Beaton A Okello E Lwabi P Mondo C McCarter R Sable C. Echocardiography screening for rheumatic heart disease in Ugandan schoolchildren. Circulation (2012) 125(25):3127–32. 10.1161/CIRCULATIONAHA.112.092312 22626741

[26] Tessema T Abuohay M Congenital malformations in Gondar Hospital, Ethiopia. East Afr Med J (1995) 72(8):495–7. 7588142

[27] Kamdem F Kedy Koum D Hamadou B Yemdji M Luma H Doualla MS Clinical, echocardiographic, and therapeutic aspects of congenital heart diseases of children at Douala General Hospital: a cross-sectional study in sub-Saharan Africa. Congenit Heart Dis (2018) 13(1):113–7. 10.1111/chd.12529 28871660

[28] Puri K Kazembe P Mkaliainga T Chiume M Cabrera AG Sims Sanyahumbi A. Pattern of inpatient pediatric cardiology consultations in sub-Saharan Africa. Congenit Heart Dis (2018) 13(2):334–41. 10.1111/chd.12573 29372615

[29] Moses AE Goldberg S Korenman Z Ravins M Hanski E Shapiro M Invasive group A streptococcal infections. Israel Emerg Infect Dis (2002) 8(4):421–6. 10.3201/eid0804.010278 11971778 PMC2730245

[30] Shoham AB Haklai Z Dor M Bar-Meir M Rheumatic fever and Kawasaki disease among children in Israel. Harefuah (2014) 153(12):709–12. 25654910

[31] Chiobotaru P Yagupsky P Fraser D Dagan R. Changing epidemiology of invasive *Streptococcus pyogenes* infections in southern Israel: differences between two ethnic population groups. Pediatr Infect Dis J (1997) 16(2):195–9. 10.1097/00006454-199702000-00006 9041600

[32] Zuhlke L Karthikeyan G Engel ME Rangarajan S Mackie P Cupido-Katya Mauff B Clinical outcomes in 3343 children and adults with rheumatic heart disease from 14 low- and middle-income countries: two-year follow-up of the global rheumatic Heart Disease Registry (the REMEDY Study). Circulation (2016) 134(19):1456–66. 10.1161/CIRCULATIONAHA.116.024769 27702773

[33] Zuhlke L Engel ME Karthikeyan G Rangarajan S Mackie P Cupido B Characteristics, complications, and gaps in evidence-based interventions in rheumatic heart disease: the Global Rheumatic Heart Disease Registry (the REMEDY study). Eur Heart J (2015) 36(18):1115a–22a. 10.1093/eurheartj/ehu449 25425448 PMC4422972

